# Note on Scurvy as a Cause of Hæmaturia in the Infant and in Old Age

**Published:** 1903-12

**Authors:** R. Shingleton Smith

**Affiliations:** Senior Physician, Bristol Royal Infirmary; Emeritus Professor of Medicine, University College, Bristol.


### NOTE ON SCURVY AS A CAUSE OF HÆMATURIA IN THE INFANT AND IN OLD AGE.

BY

R. Shingleton Smith, M.D., B.Sc. Lond., F.R.C.P.,

*Senior Physician, Bristol Royal Infirmary; Emeritus Professor of Medicine, University College, Bristol.*

Two cases have recently been under my observation in which hæmaturia has been the leading feature: the one patient was an infant, the other at the opposite extreme of life, 87; both were fed on sterilised milk and proprietary foods. The hæmaturia in both was clearly of scorbutic origin, and in both the hemorrhage rapidly subsided on the administration of an anti-scorbutic diet.

The infant was first seen on August 31st, 1903, in consequence of weakness of the lower limbs and something wrong with the urine. He was born at Christmas, and was fed artificially from the time of birth. The parents were strong and in good health, and the baby looked equally so up to about six months after birth; its weight at birth was seven pounds. It grew and appeared to prosper on a well-known proprietary infants' food. This food was made up with Pasteurised milk diluted with half water. No other food was given as the baby was well nourished, and the symptoms did not appear to be dependent on feeding or malnutrition. In August the mother took the baby to Bournemouth, and whilst there consulted a doctor, who examined the urine and found it to be albuminous. A few weeks before this the urine had been examined and was then said to be normal; but at this time (July) the mother had noticed something like blood in the urine. At the end of August there was definite and decided hæmaturia, but there was no œdema and no other evidence of any kidney defect: no casts could be found in the urinary sediment.

The teething began at about three months and continued normally; but the urine was at no time normal after six months from time of birth, and it was at this time that the weakness and soreness of legs became pronounced.

At Bournemouth in August the legs were always so tender that the child lay in an immobile state with the legs supported on a pillow, and screamed on the slightest touch or movement; but there was no actual paralysis and no appearance of wasting: the arms do not appear to have been affected, but the back was feeble.

Early in September the urine was constantly tinged with blood, and at this time there was some discolouration of and bleeding from the gums. It was then suspected that the hæmaturia and the weakness of the legs might be of scorbutic origin and due to some defect in the food; accordingly a change was made in the feeding. Fresh milk, unsterilised, was substituted for the sterilised milk in the preparation of the food; the juice of an orange was given, and was taken readily. No medicine was required, for in a few days after the change in diet the baby showed decided improvement, the tenderness of the legs became less pronounced, and the hæmaturia diminished. In the course of a week or so the urine had resumed its natural colour, although it still contained an appreciable amount of albumin, and the legs were resuming a more normal condition.

At the present time (October 25th) the baby is absolutely well, with the exception of a faint trace of albumin in the urine.

There is no tenderness of the limbs, which are used actively and with pleasure, and the teething is progressing normally. There is no manifest deformity or defect of any of the bones or joints, or any other evidence of rickets.

This case is a somewhat exceptional one, inasmuch as the leading features of infantile scurvy-rickets were not very decided, although there can be no doubt that the hæmaturia and inability of the lower limbs was of scorbutic origin.

The other case, that of a man 87 years of age, may be described as one of senile purpura with hæmaturia. He had been an invalid for months with feeble heart and anasarca of the limbs. Purpuric patches had appeared from time to time on the dorsum of the hands and also on the œdematous legs; the digestive powers were feeble, so that sterilised or Pasteurised milk, with or without Benger's food, was the principal variety of diet. On the occurrence of the hæmaturia a change in the diet was made: a vegetable soup was given, and the patient was encouraged to take a little potato with gravy, and some fresh meat juice; he also drank the juice of an orange and sucked some grapes. The change in the diet was soon followed by a cessation of the hæmaturia, and an improvement in the general state of the patient soon followed, for the anasarca became very considerably less, the power and action of the heart increased, and the patient—before apparently dying from failing heart and uræmic drowsiness—revived to such an extent that he has since had and recovered from an attack of pneumonic consolidation of the lower lobe of one lung. There has been no return of the hæmaturia since the change in the diet was made.

Since the year 1875, when attention was first directed to the subject of scurvy in infants, much has been written by Gee, Cheadle, Barlow and others; nevertheless, the condition may still be easily overlooked when it occurs amongst the well-to-do class of patients who are able to afford to buy the more expensive proprietary foods, and whose infants get less variety in their feeding than is the case with our hospital patients, amongst whom the condition is somewhat rare. The sub-periosteal hemorrhages, to which the pseudo-paralysis is due, have been fully described, and there is no necessary connection with either rickets, syphilis or rheumatism.

This case shows that the hæmaturia may be almost the only manifestation of scurvy in the infant; and its early recognition is of importance, in order that the necessary change of diet may be adopted without undue delay.

The latter case also suggests the probability that some of the hæmaturia at other than the infantile period of life may be due to a cause which is easily overlooked because of its unusual occurrence: a scorbutic hæmaturia has long been recognised, and the purpuric finale of marasmus due to other causes than scurvy must of course be admitted; but it seems that our chronic invalids who are limited to a milk diet, and who take no other nourishment than that which is cooked, are running a risk which is often forgotten and ignored.

There is no evidence that sterilised milk is itself the cause of scurvy, nor have we any reason to think that Pasteurised milk predisposes thereto; but the persistent deprivation of fresh food at any period of life clearly entails the risk of a scorbutic hemorrhage as one of the earliest indications that a change in the diet is needful.

So also the routine feeding of tender infants upon the manufactured products of commercial firms should be watched with caution; and doubtless it is due to this cause that cases of so-called scurvy-rickets have been so much more frequent in America, where the proprietary foods have been much more in use than in this country.

It is to be regretted that the cooking of all foods of this kind should deprive them of some of their anti-scorbutic powers; nevertheless, we cannot do without them, and we cannot afford in towns to dispense with the process of Pasteurisation or sterilisation of the milk which comes to us contaminated with pathogenic germs either from accidental introduction or from the cow herself.

